# Silicon Is Linked to Tea Quality Through Alteration Aluminum Uptake and Translocation in *Camellia sinensis* L.

**DOI:** 10.3390/foods14223966

**Published:** 2025-11-19

**Authors:** Hui Liu, Dongyang Wang, Yunting Bian, Wenzhe Bao, Yuanhui Hu, Chunrong Chang

**Affiliations:** School of Tropical Agriculture and Forestry, Hainan University, Haikou 570100, China; liuhuiahead@163.com (H.L.); w13017452975@163.com (D.W.); m13903370206@163.com (Y.B.); bwz19980603@163.com (W.B.); 18015178313@163.com (Y.H.)

**Keywords:** silicon, aluminum distribution, tea (*Camellia sinensis*), quality, uptake rate

## Abstract

Aluminum (Al) is involved in almost all aspects of abiotic stress and is supposed to play a crucial role in tea quality. Both exogenous Al and silicon (Si) influence Al uptake, translocation, and accumulation in considerable quantify plants. However, the relationship between Si-mediated Al uptake and tea quality has not been systematically investigated. In this study, we found that Si supply affected caffeine, free amino acids, and Al in young leaves and tea infusion. Si increased the Al content in secondary roots, but it also decreased the ratio of young leaves to secondary roots, with this decreasing ratio becoming more pronounced at higher Al supply levels. Furthermore, Si reduced the Al absorption rate at a constant pH and down-regulated the value of the Michaelis constant (Km). These analyses demonstrate that Si regulates the Al absorption rate and distribution in tea plants—that is, the ratio of Al content in young tea leaves to that in the secondary roots, thereby influencing the concentrations of caffeine, free amino acid, and Al solubility in tea infusions.

## 1. Introduction

Tea plants have been cultivated in China for over 2000 years. However, a notable characteristic of the tea plant is its accumulation of aluminum (Al) from soil. Al in tea leaves can be transferred into tea infusions through brewing. Consequently, drinking tea infusions with high Al levels may pose a potential threat to human health [1,2,3]. The United States Environmental Protection Agency (EPA) proposed a standard reference dose (RfD) for Al as 1000 μg/kg/day [4]. In a risk assessment of different tea types on the market, the contribution of Al in black and green tea was as high as 3.33–15.8%. Therefore, Al accumulation should be avoided during tea cultivation and processing [5]. With the increasing demand for premium-quality tea, the development of sustainable and efficient strategies for enhancing tea quality while reducing the Al content has become a critical focus of both tea research and production. Al is one of the most important environmental variables affecting tea plant growth and quality. As the age of tea gardens increases, the soil pH decreases by 0.47–1.43, and the soil pH is significantly negatively correlated with active Al in the soil [6]. High active Al has adverse effects on tea quality, such as reducing key tea quality traits, such as the caffeine and amino acid contents, and increasing the Al content in tea leaves [7]. Reducing the Al content in tea while improving its quality has become a purpose of tea research. In addition, silicon (Si) and Al can become hydroxyaluminosilicates [8]. The degree and direction of these effects are closely related to the concentrations of Si and Al in the environment [9,10,11,12].

In plants, Si modulates the distribution, concentration, and speciation of Al, but its effectiveness depends on the exogenous Al and Si concentrations in the growth medium and the plant species. For example, Lima et al. [13] reported a 46.04% decrease in the Al content in eucalyptus leaves after 30 days of treatment with 1.6 mM Al and 2.0 mM Si, compared to the Al-only control. Similar reductions in the Al content have been observed with Si application in rice [14], peanut [15], and teosinte [16]. Conversely, Rahman et al. [17] observed that exposure to 5 μM Al and 2.0 mM Si for 5 days increased Al accumulation in rice roots and shoots by 1.07–1.15- and 0.93–2.00-fold, respectively, compared to seedlings receiving only Al. Xiao et al. [18] found that 1.0 mM Si supplementation under 50 μM Al stress did not alter the symplastic Al content in root tips over 24 h but significantly reduced the apoplastic Al content compared to the Al-only control. Cocker et al. [19] reported that 3-day-old wheat seedlings exposed to 0.10 mM Al and 2.0 mM Si for 4 days at pH 4.2 and 4.6 showed no significant difference in root Al accumulation compared to identical Al concentrations without Si. These inconsistencies show the contextual dependencies of the effects of Si. However, the influence of Si on Al accumulation in tea plants has been rarely studied.

Tea quality is mainly determined by the free amino acid and caffeine contents. The free amino acid content is positively correlated with tea quality and has been shown to protect the nerves and relieve stress [20,21]. Caffeine is closely related to the quality of black tea and has diuretic and cardiovascular protective effects on human health [22,23]. Previous studies have shown that tea quality is influenced by the Si concentration. For example, Yang et al. [24] demonstrated that a single foliar spray of 500 mg/L Si fertilizer (Na_2_SiO_3_·9H_2_O) increased Si accumulation in the first–fifth leaves, stems, and roots and the amino acid content by 15.06–94.48% and reduced the caffeine content by 7.07–32.91% compared to non-sprayed controls. Xia et al. [25] observed that foliar application of 1000 mg/L Si (Na_2_SiO_3_·15H_2_O) enhanced the amino acid and caffeine contents in spring tea by 33.47 and 25.50% and that soil application of Si had no significant effect on the amino acid content in summer tea or the caffeine content in autumn tea, although the caffeine content was significantly reduced in summer tea. They also observed that fluctuations in the amino acid content in autumn tea were dependent on the applied Si concentration compared to treatments without Si. Wang et al. [26] reported an 11.4% increase in the caffeine content and a 12.4% increase in the amino acid content following the application of 0.66% Si seaweed fertilizer, compared to plantations treated with clean water. However, the effects of Si application under Al stress on tea quality in a hydroponic environment have been rarely studied.

However, the mechanisms by which Si influences Al uptake and distribution in tea and the effects of Si application under Al stress remain unknown. Further investigation is needed to determine the effects of Si application on Al uptake and tea quality. Therefore, the present study aimed to determine the effects of Si application on Al uptake and distribution of Al and tea quality under a constant pH and Al species. This study provides a scientific basis for rational Si application in tea cultivation practices.

## 2. Materials and Methods

### 2.1. Plant Materials and Treatments

Tea seeds (*Camellia sinensis* var. *assamica* cv. ‘Hainan Dayezhong’) were obtained from Wuzhishan Sanrui Daye Tea Industry Co., Ltd. (Wuzhishan City, Hainan Province, China). After removing the husk, the seeds were surface-sterilized following a previously described method [27]. The seeds were then planted in a sand bed at the Agricultural Science Base of Haidian Campus, Hainan University, Haikou City, Hainan Province. Plants were cultivated from November 2022 to July 2023. When the seedlings developed one bud and four leaves, they were transferred and grown hydroponically in half-strength nutrient solution [28] in a greenhouse under natural light conditions. There were four plastic pots (length: 56 cm; width: 38 cm; height: 15 cm), each containing 24 seedlings. The solution was continuously aerated at a rate of 1.5 L/min for 12 h per day. All chemical reagents [28] used in this study were analytically pure. The plants were pre-cultured in a nutrient solution for 3 weeks to increase new root growth, and those of uniform size were used for two subsequent experiments in a greenhouse under natural conditions.

Eight-month-old seedlings were divided into the following six treatment groups and cultivated for 5 weeks: −Si−Al (0 mM Si, 0 mM Al), +Si−Al (0.20 mM Si, 0 mM Al), −Si+Al_0.20_ (0 mM Si, 0.20 mM Al), +Si+Al_0.20_ (0.20 mM Si, 0.20 mM Al), −Si+Al_1.0_ (0 mM Si, 1.0 mM Al), and +Si+Al_1.0_ (0.20 mM Si, 1.0 mM Al). Three seedlings were placed in one 4.5 L black plastic bucket (upper mouth diameter: 28 cm; lower mouth diameter: 19 cm; depth: 15 cm) and filled with half-strength nutrient solution [28]. There were five buckets (replicates) for each treatment (Figure S1a). The nutrient solutions containing Al and Si were prepared by adding Al_2_(SO_4_)_3_·18H_2_O and Na_2_SiO_3_·9H_2_O to the hydroponic solution according to the experimental design. During cultivation, the pH of the nutrient solution was adjusted to 4.0 by adding 0.50 M NaOH or H_2_SO_4_ every day, and the solution was refreshed every week. At the end of the experiment, the plants were carefully removed from the solution, gently rinsed four times in deionized water, and surface-dried with absorbent paper (Figure S1b). The plants were separated into young leaves (one bud and two leaves), secondary roots, and primary roots (grown in sand culture). Each sample was oven-dried at 105 °C until a constant weight was achieved. Samples were then ground finely in an electric grinder and thoroughly mixed for subsequent analysis.

To confirm the effect of the presence or absence of Si on Al uptake kinetics under different Al concentrations, we set up another experiment. After the tea seedlings were grown in 0.20 mM CaSO_4_ solution (pH 4.0) for 5 days under natural light conditions, each seedling was placed in a black bottle (upper mouth diameter: 5 cm; lower mouth diameter: 8.6 cm; depth: 14 cm) containing 500 mL nutrient solution with different Al concentrations (0.035, 0.07, 0.14, 0.56, 1.12, 1.40, 1.68, and 1.96 mM), and Si concentrations were set at 0.0 and 0.20 mM. After 8 h, 500 mL of nutriment solution (without Al or Si) was added to replenish nutrients lost through water uptake. A 20 mL aliquot of the mixed solution was collected as the test sample to determine the Al content. The seedlings were carefully removed after the nutrient solution was drained. The roots were separated from each seedling using scissors, oven-dried at 105 °C, and then weighed to determine the dry weight for uptake kinetics analysis.

### 2.2. Analytical Parameters and Methods

The caffeine and free amino acid contents in young leaves were quantified using the ultraviolet spectrophotometry (UV754N, Shanghai Yidian Analysis Instrument Co., Ltd., Shanghai, China) [29] and ninhydrin colorimetric methods (722S, Shanghai Youke Instrument Co., Ltd., Shanghai, China) [30], respectively. The Al contents in tea plant tissues (young leaves, primary roots and secondary roots) and uptake solution samples were determined using the chrome azurol S colorimetric (722S, Shanghai Youke Instrument Co., Ltd., Shanghai, China) [31] and Al reagent colorimetric methods (722S, Shanghai Youke Instrument Co., Ltd., Shanghai, China) [32], respectively. The Si content in young leaves was determined following the methods described by Alamri et al. [33], Dai et al. [34], and Yang, Zhang, Cui, Hou, Xie and Han [24]. Tea infusions were produced from crushed young leaf samples, the protocol established by Bora et al. [35]. In the tea infusion, the Al content was determined using inductively coupled plasma optical emission spectrometry (Plasma 3000 ICP-OES, NCS Testing Technology Co., Ltd., Beijing, China) [36], and the Si content was determined using the colorimetric Si molybdenum blue method (722S, Shanghai Youke Instrument Co., Ltd., Shanghai, China) [37]. The Al and Si solubility in tea infusion was calculated according to the method of Oliveira et al. [38].

### 2.3. Uptake Kinetics Parameters

According to the mass change in the Al content in the solution before and after uptake, the relationship between the Al uptake rate in tea plants and the nutrient solution concentration was calculated by the plotting method with the root dry weight as the dimension [39]. The maximum uptake rate (Vmax), Michaelis constant (Km), and rate of net influx (α) were calculated using this plotting method [40].

### 2.4. Health Risk Assessment

According to the evaluation method described by Li et al. [41], the oral standard reference dose (RfD) for Al was set at 1000 μg/kg/day. The daily tea infusion consumption was assumed to be 1250 mL/person/day (F_IR_), and the average adult body weight was set as 60 kg [42,43]. The potential health risk associated with Al exposure was evaluated using the estimated daily intake (EDI) and the target hazard quotient (THQ), which were calculated according to the method described by Li, Fu, Achal and Liu [41].

### 2.5. Data Processing and Statistical Analysis

Raw data were compiled and statistically analyzed using Microsoft Excel 2019 (Microsoft Corp., Redmond, WA, USA). One-way analysis of variance (ANOVA) was conducted using SPSS 22.0 software (IBM, Amonk, NY, USA). Differences between mean values were determined using the least significant difference (LSD) test at *p* < 0.05. Origin software (version 2022, Origin Lab Corp., Northampton, MA, USA) was used to perform Pearson correlation coefficient analysis and generate plots.

## 3. Results

### 3.1. Caffeine and Free Amino Acid Contents in Young Tea Leaves

**Figure 1:**
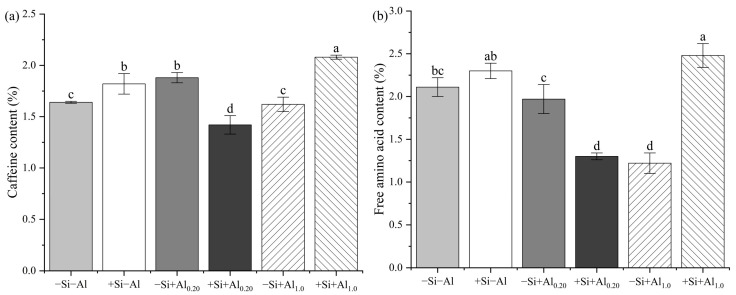
Quality components affected by exogenous silicon (Si) and aluminum (Al) application in tea plants: (**a**) caffeine content; (**b**) total free amino acid content. Bars represent the mean ± standard deviation, *n* = 5. Different lowercase letters above the bars indicate significant differences between treatments according to the LSD test (*p* < 0.05). Treatments: −Si−Al: 0 mM Si, 0 mM Al; +Si−Al: 0.20 mM Si, 0 mM Al; −Si+Al_0.20_: 0 mM Si, 0.20 mM Al; +Si+Al_0.20_: 0.20 mM Si, 0.20 mM Al; −Si+Al_1.0_: 0 mM Si, 1.0 mM Al; +Si+Al_1.0_: 0.20 mM Si, 1.0 mM Al.

The effects of Si supply on the caffeine and free amino acid contents in young tea leaves were investigated under Al application (Figure 1). The influence of Si on the caffeine and free amino acid contents in young tea leaves was associated with the Al concentration in the nutrient solution. The regulation of caffeine and free amino acid contents by Si increased, decreased, and increased under 0, 0.20, and 1.0 mM Al concentrations, respectively. Treatment with +Si+Al_0.20_ increased the caffeine and free amino acid contents in young leaves by 24.47 and 34.01%, respectively, compared to −Si+Al_0.20_ treatment. The application of Si increased the caffeine and free amino acid contents in young leaves by 22.12 and 50.81%, respectively, compared to the treatment with only 1.0 mM Al. Without Al addition, Si supply increased the caffeine content in young tea leaves by 10.98%, but the free amino acid content did not significantly change.

Variations in the caffeine and free amino acid contents were analyzed under different Al concentrations. The caffeine content in young tea leaves increased by 14.6% under −Si+Al_0.20_ treatment compared to that under −Si−Al treatment; however, there was no significant difference in the free amino acid content. In contrast, the −Si+Al_1.0_ treatment reduced the free amino acid content in young tea leaves by 73.0% compared to the −Si−Al treatment, but there was no significant effect on the caffeine content.

### 3.2. Al and Si Content in Young Tea Leaves

**Figure 2:**
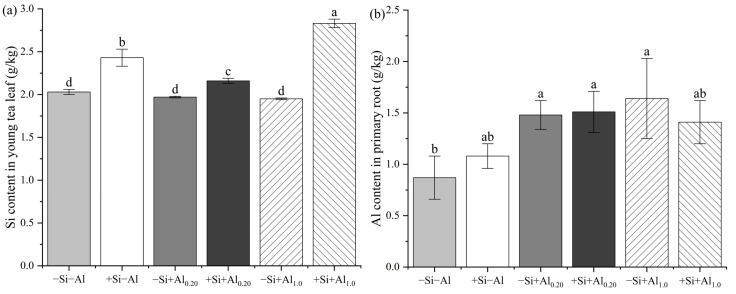
Effects of exogenous aluminum (Al) and silicon (Si) application on the (**a**) Si content in young tea leaf, and (**b**) Al content in primary root. Bars represent the mean ± standard deviation, n = 5. Different lowercase letters above the bars indicate significant differences between treatments according to the LSD test (*p* < 0.05). Treatments: −Si−Al: 0 mM Si, 0 mM Al; +Si−Al: 0.20 mM Si, 0 mM Al; −Si+Al_0.20_: 0 mM Si, 0.20 mM Al; +Si+Al_0.20_: 0.20 mM Si, 0.20 mM Al; −Si+Al_1.0_: 0 mM Si, 1.0 mM Al; +Si+Al_1.0_: 0.20 mM Si, 1.0 mM Al.

As shown in Table 1 and Figure 2, the Si content in young tea leaves increased with the Si application under the same Al dosage. The increase in the Si content in young tea leaves was correlated with increased Al application. Si treatment significantly reduced the Al content in young tea leaves in the absence of Al, showing an inverse relationship with Al addition. The Al content decreased by 20.69% in young tea leaves subjected to +Si−Al treatment compared to −Si−Al treatment. Young tea leaves from seedlings under Si treatment had a higher Al content (+24.14%) than those without Si under 0.20 mM Al treatment. Exogenous Si did not significantly affect the Al content in young tea leaves when the seedlings were treated with 1.0 mM Al. In the absence of Si, the Al content in young tea leaves was nearly twice as high when Al was present compared to the treatment without Al. Compared to the −Si treatment, the Si content increased by 19.7, 9.60, and 45.1% under +Si treatments with Al (0, 0.2, and 1.0 mM, respectively). The effect of the Al concentration on the Si content was not significant without Si application.

### 3.3. Al Content in Primary and Secondary Roots of Tea Seedlings

As shown in Table 1, Si treatment significantly increased the Al content in the secondary roots of tea seedlings under the same applied Al concentration. The effect of Al was more pronounced with increasing exogenous Al concentrations. In contrast, no significant differences in the Al content were observed in the primary roots across all six treatments (Figure 2b). Compared to the −Si treatments, +Si treatments with 0.20 and 1.0 mM Al increased the Al content in the secondary roots by 10.0 and 20.8%, respectively. The Al content in the secondary roots increased by 97 and 153%, while in the primary roots, it increased by 70.1 and 88.5%, respectively, with the application of 0.20 and 1.0 mM Al compared to the −Al treatment. The Al content in both roots and young leaves was related to the applied Al concentration. Si increased the Al content in secondary roots, but it decreased the Al content in young leaves when higher Al concentrations were applied in the nutrient solution. The ratio of the Al content in young tea leaves to that in secondary roots was 0.44, 0.24, and 0.46 for +Si+Al_0.2_, +Si+Al_1.0_, and +Si−Al treatments, respectively (Table 1).

### 3.4. Al and Si Contents and Solubility in the Tea Infusion

**Figure 3:**
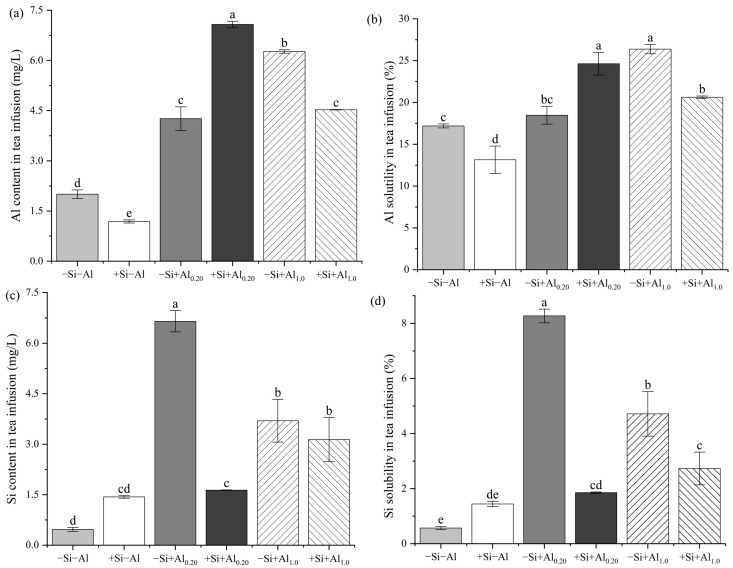
Effects of exogenous aluminum (Al) and silicon (Si) application on the content and solubility of Si and Al in the tea infusion: (**a**) Al content in the tea infusion, (**b**) Al solubility in tea infusion, (**c**) Si content in tea infusion, and (**d**) Si solubility in tea infusion. Bars represent the mean ± standard deviation, *n* = 5. Different lowercase letters above the bars indicate significant differences between treatments according to the LSD test (*p* < 0.05). Treatments: −Si−Al: 0 mM Si, 0 mM Al; +Si−Al: 0.20 mM Si, 0 mM Al; −Si+Al_0.20_: 0 mM Si, 0.20 mM Al; +Si+Al_0.20_: 0.20 mM Si, 0.20 mM Al; −Si+Al_1.0_: 0 mM Si, 1.0 mM Al; +Si+Al_1.0_: 0.20 mM Si, 1.0 mM Al.

The effect of Si on the Al and Si contents in the tea infusion was associated with the applied Al concentration (Figure 3a,c). The Al content in the tea infusion was reduced by 40.5% under +Si−Al compared to −Si−Al and 27.8% under +Si+Al_1.0_ compared to −Si+Al_1.0_. Conversely, the Al content in the tea infusion increased by 66.2% under +Si+Al_0.20_ compared to −Si+Al_0.20_. Exogenous Si increased the Si content in the tea infusion by 206% compared to the −Si treatment when no Al was applied. However, when 0.20 mM Al was applied, Si application reduced the Si content in the tea infusion by 75.3% compared to the treatment without Si application. There were no significant differences in the Si content in the tea infusion between +Si and −Si treatments with 1.0 mM Al.

The solubilities of Al and Si in the tea infusion were consistent with the variations in their contents within the tea infusion, particularly under the same applied Al concentration. The solubilities of Al and Si in the tea infusion were influenced by the applied Al concentration. Notably, there were no significant effects of the Al concentration applied with Si on the Si solubility in the tea infusion (Figure 3b,d). The Al solubility was reduced by 23.5% under +Si−Al compared to −Si−Al and by 21.8% under +Si+Al_1.0_ compared to −Si+Al_1.0_. Conversely, under +Si+Al_0.20_, the Al solubility increased by 38.8% compared to that under −Si+Al_0.20_. Compared to the −Si treatments with the same applied Al concentration, Si application reduced the Si solubility by 77.5 and 42.0% with 0.20 mM and 1.0 mM Al supply, respectively, while it was increased by 153% in the absence of Al.

### 3.5. Effects of Si Supply on Al Uptake in Tea Seedlings

**Figure 4:**
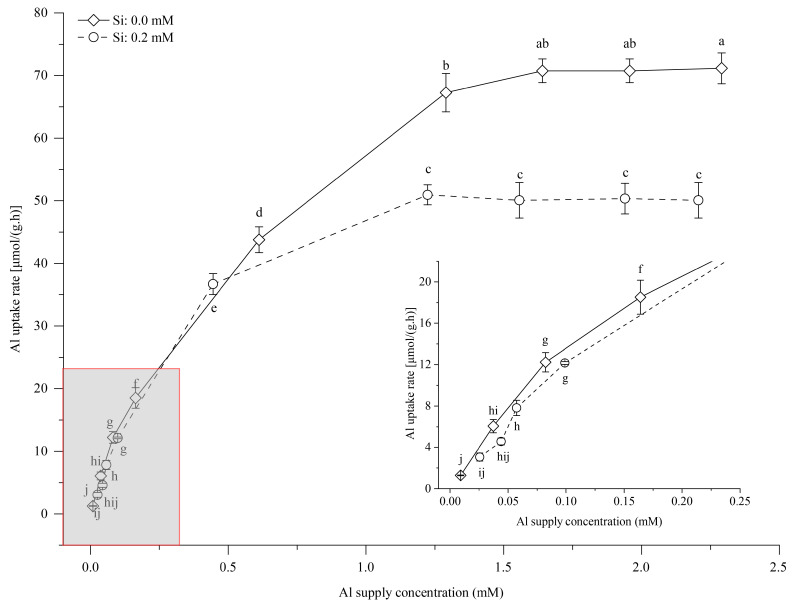
Aluminum (Al) uptake kinetic curves of tea seedlings under two silicon (Si) application treatments. Bars represent the mean ± standard deviation, *n* = 5. Different lowercase letters above (Si: 0.0 mM) and below (Si: 0.2 mM) the data points indicate significant differences in the Al uptake rate between Al concentrations under Si supply according to the LSD test (*p* < 0.05).

The Al concentration uptake rate curves in tea seedlings under +Si and −Si conditions are shown in Figure 4. Both the +Si and −Si curves exhibited relatively low uptake rates when the applied Al concentration ranged from 0.035 to 0.28 mM, but this was followed by a sharp increase as the applied Al concentration rose from 0.28 to 1.0 mM and a gradual increase when it ranged from 1.0 to 1.96 mM. These trends conform to the Michaelis–Menten enzyme kinetics model. The presence of Si significantly reduced the Al uptake rate by 16.2–36.1% at applied Al concentrations from 0.035 to 1.96 mM, compared to treatment without Si.

The coefficients of the Al uptake kinetic equation and the uptake characteristics in tea seedlings showed that Al uptake in tea seedlings was significantly influenced by Si application (Table 2). The coefficient of the linear term in the kinetic equation and the maximum and average uptake rates decreased by 15.9, 25.6, and 24.7%, respectively, with the addition of Si compared to conditions without Si. The affinity coefficient increased with Si application, as evidenced by a 17.0% decrease in the Km value under the Si treatment compared to the treatment without Si.

The effects of Si application on the caffeine, free amino acid, and Al contents in young leaves and roots, as well as the Al uptake rate were comparatively analyzed. The findings showed that the increase in tea quality associated with Si was linked to the suppression of the Al uptake rate and a reduction in Al translocation from roots to young leaves. Correlations were calculated separately for the application of 0.20 and 1.0 mM Al, considering the Al content in young tea leaves, primary roots, secondary roots, and the tea infusion, as well as the Al and Si solubilities in young leaves and the Si content in young leaves and tea infusion. With 0.20 mM Al application, the Al content in young tea leaves was negatively correlated with the Si content in those leaves, although it was positively correlated with the Al content in secondary roots and the Si content in the tea infusion. The correlation results were reversed with 1.0 mM Al application.

## 4. Discussion

### 4.1. Free Amino Acid and Caffeine Contents in Young Tea Leaves

Tea plants, which are known for their ability to accumulate Al, exhibit high Al stress resistance. The Al content in tea plays a crucial role in determining its overall quality [5,7]. Al is a primary factor causing variations in the free amino acid and caffeine contents in young tea leaves, and plants can adapt to high Al environments by increasing the amino acid and caffeine contents [7]. However, some studies have shown that the amino acid content increases under a relatively low Al supply, as the Al concentration increases, the amino acid content decreases, whereas the caffeine content increases [7,44]. These responses have been associated with the tea variety and the concentration of exogenous Al applied [45]. In the present study, the free amino acid content was not significantly affected, whereas the caffeine content was significantly increased after treatment with 0.20 mM Al compared to the treatment without Al in 8-month-old Hainan Dayezhong tea seedlings. However, after applying 1.0 mM Al, the caffeine content was not significantly affected, but the free amino acid content significantly decreased. These results are consistent with those of Huang et al. [46]. The present study also showed that the caffeine and free amino acid contents in young tea leaves were influenced by Si and applied Al concentration. The caffeine and free amino acid contents in young tea leaves were significantly higher under treatments with +SiAl_1.0_ compared to +Si+Al_0.20_, −Si+Al_0.20_, and −Si+Al_1.0_. Notably, compared to all other treatments, the lowest caffeine and free amino acid contents were observed under +Si+Al_0.20_ (Figure 1). These findings are not entirely consistent with those of Xia, Yang, Li and Wu [25], who reported that foliar application of 1000 mg/L Si (Na_2_SiO_3_·15H_2_O) enhanced the amino acid and caffeine contents in spring tea by 33.47 and 25.50%, respectively. These discrepancies may be attributed to differences in the method by which Si was applied to the tea plants [26,45,47].

### 4.2. Al Content in Young Tea Leaves and Roots

The study of Al accumulation in the roots and aerial parts of plants has become a crucial area of research for the adaptation of plants to high Al environments and mitigating the toxic effects of Al [9,10,48]. However, the impact of external conditions on the Al content and distribution in tea plants has seldom been reported. Previous research has demonstrated that the Al contents in the roots and aerial parts of various plant species increase with the applied Al concentration [49]. In this study, the Al contents in primary roots, secondary roots, and young tea leaves significantly increased with 0.20 mM Al application compared to the −Al treatment. The Al content showed no significant change in young leaves or primary roots but significantly increased in secondary roots under +Al_1.0_ compared to treatment without Al (Table 1 and Figure 2). The reduction in the Al contents in roots and aerial parts via Si application has been verified among plants [50]. For example, Singh, Tripathi, Kumar and Chauhan [14] demonstrated that rice seedlings treated with 50 μM Al and 10 μM Si for 7 days exhibited reductions of 34.82 and 38.89% in the root and shoot Al contents, respectively, compared to Al-only controls. Similarly, Shen, Xiao, Dong and Chen [15] observed that peanut seedlings exposed to 0.66 mM Al and 0.28 mM Si for 7 days had 40.71 and 25.5% lower Al concentrations in their roots and leaves, respectively, compared to those treated with equivalent Al levels without Si. In our study, the Al content in young tea leaves under +Si+Al_0.20_ was higher than that under −Si+Al_0.20_, but the Al content in young tea leaves under +Si+Al_1.0_ showed no significant difference compared to that under −Si+Al_1.0_. Differences in the studies might contribute to the differences in the effects of Si on Al uptake in tea plants under different Al concentrations. Treatment with +Si+Al_0.20_ resulted in the highest Al content among all treatments. A significantly higher Al content was observed in secondary roots, but no significant change was observed in the primary roots due to increases in the applied Al concentration with a Si supply (Table 1 and Figure 2). These results are consistent with those of Fan, Wang, Gao, Ning and Shi [9] but do not agree with those of Wang, Ning and Shi [47] and Morita et al. [51].

Previous research has reported that Si and Al could become hydroxyaluminosilicates [8]. The co-localization of Al and Si can also be found in tea leaves [52]. In the present study, Si application reduced the Al content and leaching rate in young tea leaves and infusion as the applied Al concentration increased, and the effect of Si on the Al content in tea roots was mainly observed in the emerging root system. These results indicate that the hydroxyaluminosilicates may be generated in the root and leaves under Si and Al application during tea plant cultivation [52,53].

### 4.3. Si Content in Young Tea Leaves and Al Uptake Characteristics in Tea Seedlings

Si enhances plant resistance against abiotic stressors, including Al stress [11,54]. Previous studies have primarily investigated the influence of Si (as discussed in the previous section) and its distribution following its application [55]. The primary mechanism by which Si alleviates toxicity is through the increased Si content in the roots and aerial parts of plants. It has been suggested that Si transport to the shoots reduces Al accumulation or that Si promotes the precipitation of hydroxyaluminosilicate with Al in the root apoplast, thus increasing the amount of precipitate and reducing Al toxicity [18,56]. Our results showed that Si application significantly increases the Si content in young tea leaves. The Si content under −Si+Al_0.20_ was lower than in any other treatment (Figure 2), which may be related to the influence of Al uptake and the applied Si concentration [53,55]. The present study further showed that Si application had minimal effects on the Al uptake rate within an Al application range of 0.00–0.28 mM, but it was significantly reduced with 0.28–1.00 mM Al supply, decreasing the Vmax of Al uptake in tea seedlings (Table 2). These results may be associated with the influence of Si on Al uptake in tea roots [57].

### 4.4. Health Risk Assessment of Al in the Tea Infusion

**Figure 5:**
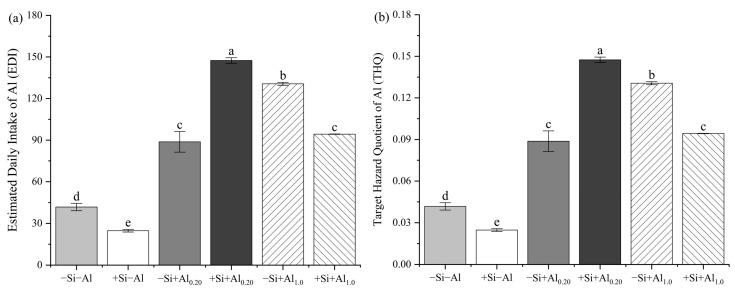
(**a**) Estimated daily intake of aluminum (Al) in tea infusions and (**b**) the target hazard quotient of Al (**b**) under Al and silicon (Si) application. The daily intake of Al in tea infusions is calculated based on the consumption of 1250 mL of tea per day. The health index of tea consumption is calculated based on the dosage standard of Al intake for tea drinkers at 1 mg/kg/day, with adult body weight assumed to be 60 kg. Bars represent the mean ± standard deviation, *n* = 5. Different lowercase letters above the bars indicate significant differences between treatments according to the LSD test (*p* < 0.05). Treatments: −Si−Al: 0 mM Si, 0 mM Al; +Si−Al: 0.20 mM Si, 0 mM Al; −Si+Al_0.20_: 0 mM Si, 0.20 mM Al; +Si+Al_0.20_: 0.20 mM Si, 0.20 mM Al; −Si+Al_1.0_: 0 mM Si, 1.0 mM Al; +Si+Al_1.0_: 0.20 mM Si, 1.0 mM Al.

As a typical Al-accumulating crop, the Al content in tea infusions is closely correlated with the Al content in young tea leaves [38]. Fung et al. [58] reported that the Al content in tea infusions ranges from 0.703 to 5.931 mg/L. In the present study, based on the calculation method of Li, Fu, Achal and Liu [41], the calculated health risk value for Al in tea infusions was far below the health risk criterion (1000 μg/kg/day [4] across all treatments (Figure 5). The Al content in young tea leaves was significantly positively correlated with the Al content and solubility in tea infusions. Correlations were analyzed separately for 0.20 and 1.0 mM Al applications to determine the relationships among the Al content in young tea leaves, the Si content in young tea leaves, the Si content in tea infusions, and the Si solubility. A negative correlation was observed between the Al and Si contents in young tea leaves, and a positive correlation was observed between the Al content in young tea leaves and both the Si content and Si solubility in the tea infusion under 0.20 mM Al application. Conversely, the opposite trend was observed under the 1.0 mM Al application. This difference suggests that the mechanisms of Al and Si uptake under varying Al supply may not be identical [17,18,19,57].

During the tea garden cultivation process, as the planting years increase, the soil pH significantly decreases by 0.47 to 1.43, which in turn increases the content of highly active Al in the soil, adversely affecting the quality of tea leaves [6,7]. Based on the results found in this work, it is recommended that (i) during the tea plantation process, it is necessary to dynamically monitor the levels of highly active Al in the tea garden soil and Al content in the young tea leaves. Based on the monitoring results, timely measures such as applying Si fertilizer should be taken to regulate the Al content in the young tea leaves, ensuring tea quality while reducing the risk of Al intake by humans through tea consumption. (ii) Due to the solubility of silicate, Si fertilizer could be dissolved in water first and then applied to the tea garden through drip irrigation or foliar spraying to reduce Al content in young tea leaves.

## 5. Conclusions

In summary, the present study focused on the effects of Si (0 and 0.20 mM) and Al application (0, 0.20, and 1.0 mM) on the Al and Si content in young tea leaves and tea infusions, the Al content in primary and secondary roots, and tea quality traits during a 35-day hydroponic cultivation experiment. Differences in Al uptake characteristics of tea seedlings were observed with 0.20 mM Si application compared to those without Si. The application of Si decreased the Al uptake rate in tea seedlings at the same applied Al concentration and reduced the Vmax of Al by 25.6% under Si treatment compared to seedlings without Si application. These results may be attributed to the influence of Si application on Al uptake. Exogenous Si increased the Al content in the primary roots and reduced Al translocation from the roots to young leaves. This was improved by increasing the applied Al concentration, which affected the caffeine, free amino acid, and Al contents in the tea infusion. Notably, the caffeine and free amino acid contents were higher when 0.20 mM Si and 1.0 mM Al were applied. The mechanisms of Al and Si uptake under different applied Al concentrations may differ, and further research is required to investigate these differences. This study provides a strategy for decreasing the Al intake risk of tea infusions through rational Si application and offers valuable insights that could significantly impact both tea cultivation practices and the understanding of tea quality formation.

## Figures and Tables

**Table 1:** The Al content in young tea leaf, primary root, and the ratio between them.

Treatment	Al Content in Young Tea Leaf (g/kg)	Al Content in Secondary Root (g/kg)	The Ratio of the Al Content in Young Tea Leaf to that in Secondary Root
−Si−Al	0.29 ± 0.01 c	0.76 ± 0.10 d	0.39 ± 0.03 ab
+Si−Al	0.23 ± 0.02 d	0.51 ± 0.09 d	0.46 ± 0.12 a
−Si+Al_0.2_	0.58 ± 0.01 b	1.50 ± 0.22 c	0.39 ± 0.05 ab
+Si+Al_0.2_	0.72 ± 0.05 a	1.65 ± 0.01 bc	0.44 ± 0.03 ab
−Si+Al_1.0_	0.59 ± 0.02 b	1.92 ± 0.09 b	0.31 ± 0.01 bc
+Si+Al_1.0_	0.55 ± 0.00 b	2.32 ± 0.08 a	0.24 ± 0.01 c

Bars represent the mean ± standard deviation, *n* = 5. Different lowercase letters above the bars indicate significant differences between treatments according to the LSD test (*p* < 0.05). Treatments: −Si−Al: 0 mM Si, 0 mM Al; +Si−Al: 0.20 mM Si, 0 mM Al; −Si+Al_0.20_: 0 mM Si, 0.20 mM Al; +Si+Al_0.20_: 0.20 mM Si, 0.20 mM Al; −Si+Al_1.0_: 0 mM Si, 1.0 mM Al; +Si+Al_1.0_: 0.20 mM Si, 1.0 mM Al.

**Table 2:** Aluminum (Al) uptake kinetic equation and its kinetic parameters in tea seedlings under silicon (Si) treatment.

Si Supply Level (mM)	Kinetic Equation	R^2^	Vmax [μmol/(g·h)]	Km (mM)	α [L/(g·h)]
0.00	y = −20.23*x*^2^ + 74.63*x* + 4.294	0.9950 **	73.12 ± 1.59 a	0.500 ± 0.001 a	0.146 ± 0.003 a
0.20	y = −19.79*x*^2^ + 62.78*x* + 4.590	0.9697 **	54.42 ± 0.21 b	0.415 ± 0.016 b	0.131 ± 0.006 b

The uptake kinetic equation presented in the table is dimensioned by root dry weight and was determined using the plotting method (*n* = 5). In the equation, y represents the uptake rate [μmol/(g·h)], and *x* represents the Al concentration in the nutrient solution (mM). ** indicates that the equation exhibits a highly significant correlation (*p* < 0.01). Different lowercase letters within a column indicate significant differences between treatments according to the LSD test (*p* < 0.05).

## Data Availability

The original contributions presented in the study are included in the article/Supplementary Materials, further inquiries can be directed to the corresponding author.

## Supplementary Materials

The following supporting information can be downloaded at https://www.mdpi.com/article/10.3390/foods14223966/s1: Figure S1. (a) The process of cultivating tea seedlings in nutrient solution; (b) the tea seedlings af-ter the cultivation experiment.

## Abbreviations

The following abbreviations are used in this manuscript:

Al	Aluminum
ANOVA	Analysis of variance
EDI	Estimated daily intake
EPA	Environmental Protection Agency
LSD	Least significant difference
Si	Silicon
THQ	Target hazard quotient
RfD	oral reference dose
Vmax	Maximum uptake rate
Km	Michaelis constant
α	rate of net influx
