# Supplementary material for: Silicon Is Linked to Tea Quality Through Alteration Aluminum Uptake and Translocation in Camellia sinensis L
Source: Foods. 2025 Nov 19;14(22):3966. doi: 10.3390/foods14223966 (PMC12652145; doi:10.3390/foods14223966)
Supplement: Supplementary file 1 [file foods-14-03966-s001.zip › foods-3969763-supplementary.pdf]

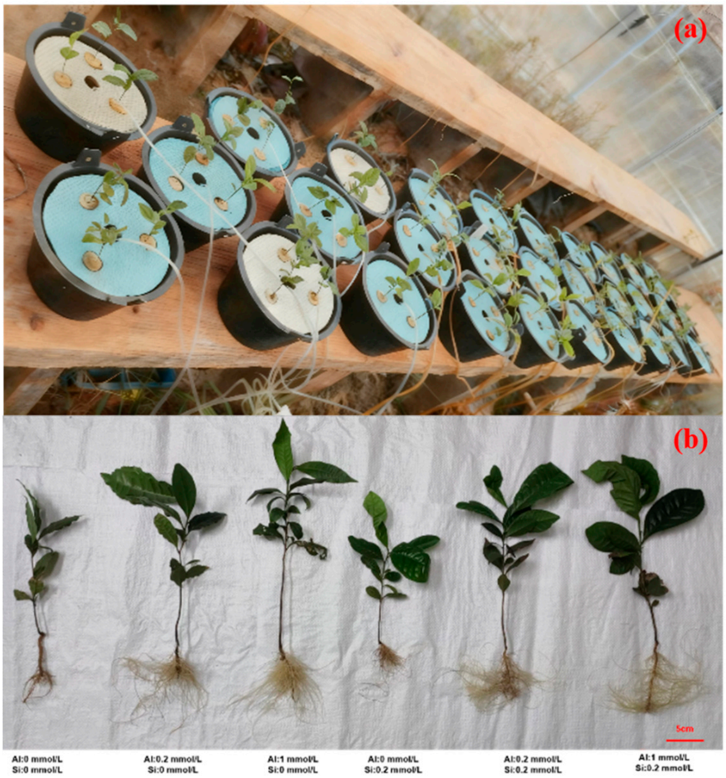

Figure S1. (a) The process of cultivating tea seedlings in nutrient solution; (b) the tea seedlings after the cultivation experiment.
